# Spatial Transcriptomics Reveals HER2/AR-Associated Heterogeneity and Alternative Surface Targets in Salivary Duct Carcinoma

**DOI:** 10.3390/ijms27209018

**Published:** 2026-10-12

**Authors:** Yoh-ichiro Iwasa, Kentaro Hori, Tomoyuki Nakajima, Yoh Yokota, Shintaro Yamazaki, Yukiko Hori, Takeshi Uehara, Yutaka Takumi

**Affiliations:** 1 Department of Otorhinolaryngology-Head and Neck Surgery, School of Medicine, Shinshu University, 3-1-1, Asahi, Matsumoto City 390-8621, Japan; 2 Department of Laboratory Medicine, School of Medicine, Shinshu University, 3-1-1, Asahi, Matsumoto City 390-8621, Japan

**Keywords:** salivary duct carcinoma, spatial transcriptomics, HER2, androgen receptor, therapeutic surface targets

## Abstract

Salivary duct carcinoma (SDC) is an aggressive malignancy primarily treated with human epidermal growth factor receptor 2 (HER2)- and androgen receptor (AR)-directed therapies in the recurrent or metastatic setting, although therapeutic responses remain heterogeneous. We used spatial transcriptomics to characterize intratumoral heterogeneity and to identify alternative therapeutic targets. Spatial transcriptomic profiling was performed on four formalin-fixed, paraffin-embedded SDC specimens using the 10× Genomics Visium platform. Tumor regions were characterized according to spatial HER2 and AR expression and their corresponding transcriptional signatures. Candidate cell-surface targets in HER2/AR-non-associated regions were identified using the SURFY human surfaceome and prioritized based on low expression in matched normal salivary gland tissue and therapeutic relevance. Spatial mapping revealed heterogeneous distributions of HER2- and AR-associated transcriptional states within individual tumors, including HER2/AR-non-associated regions in all four specimens. Surfaceome screening identified several candidate targets with low expression in matched normal salivary gland tissue, among which ALCAM showed consistently broad whole-tumor coverage across all four specimens. These findings highlight substantial intratumoral heterogeneity in SDC and identify alternative cell-surface targets that may complement HER2- and AR-directed therapies.

## 1. Introduction

Head and neck cancer (HNC) is the sixth most common malignancy worldwide and comprises a biologically and anatomically heterogeneous group of tumors arising from multiple head and neck subsites, including the salivary glands [1]. Salivary gland carcinomas (SGCs) account for approximately 5% of all HNCs and represent a rare group of malignancies characterized by marked histological diversity, with >20 subtypes recognized in the current World Health Organization classification [2]. Although squamous cell carcinoma (SCC) is the predominant histological subtype of HNC, SGCs comprise a diverse group of predominantly non-SCC histologies. Their rarity and biological heterogeneity have limited the development of standardized therapeutic strategies.

Salivary duct carcinoma (SDC) is a highly aggressive subtype of SGC characterized by a high propensity for distant metastasis and poor clinical outcomes. Surgical resection remains the standard treatment for localized disease; however, recurrent or metastatic disease frequently develops despite successful locoregional control.

SDC frequently expresses human epidermal growth factor receptor 2 (HER2) and androgen receptor (AR), with expression rates of approximately 30–40% and 70–90%, respectively [3]. HER2-targeted therapy with trastuzumab plus docetaxel for HER2-positive tumors and combined androgen blockade (CAB) for AR-positive tumors have demonstrated promising efficacy in recurrent or metastatic SDC [4,5]. Nevertheless, these treatments do not benefit all patients, and effective therapeutic options for refractory disease remain limited. Furthermore, immune checkpoint inhibitors (ICIs), which have become a key treatment modality for head and neck SCC (HNSCC), have demonstrated limited clinical efficacy in SDC [6].

Recent advances in spatial transcriptomics (ST), including Visium-based platforms, have enabled comprehensive characterization of spatially resolved gene expression within tumor tissues [7]. By preserving tissue architecture, these technologies facilitate the investigation of intratumoral heterogeneity and the tumor microenvironment. Recently, ST analysis has been applied to SDC to investigate stromal biology, particularly cancer-associated fibroblast (CAF)-associated spatial niches [8]. However, the spatial organization of HER2- and AR-associated transcriptional programs within tumor cells remains unclear.

Although conventional HER2-targeted therapies have primarily been used for HER2-positive SDC, recent evidence from the phase II MYTHOS trial suggests that T-DXd may extend HER2-directed treatment to HER2-low SDC [9,10]. Nevertheless, the spatial relationships among HER2 expression, AR-associated transcriptional programs, and potentially targetable tumor regions remain poorly understood.

Accordingly, we performed ST profiling of SDC to characterize the spatial heterogeneity of HER2- and AR-associated transcriptional states and to identify conserved cell-surface antigens with potential as therapeutic targets across heterogeneous tumor regions.

## 2. Results

### 2.1. Clinicopathological Characteristics of SDC Samples

**Figure 1:**
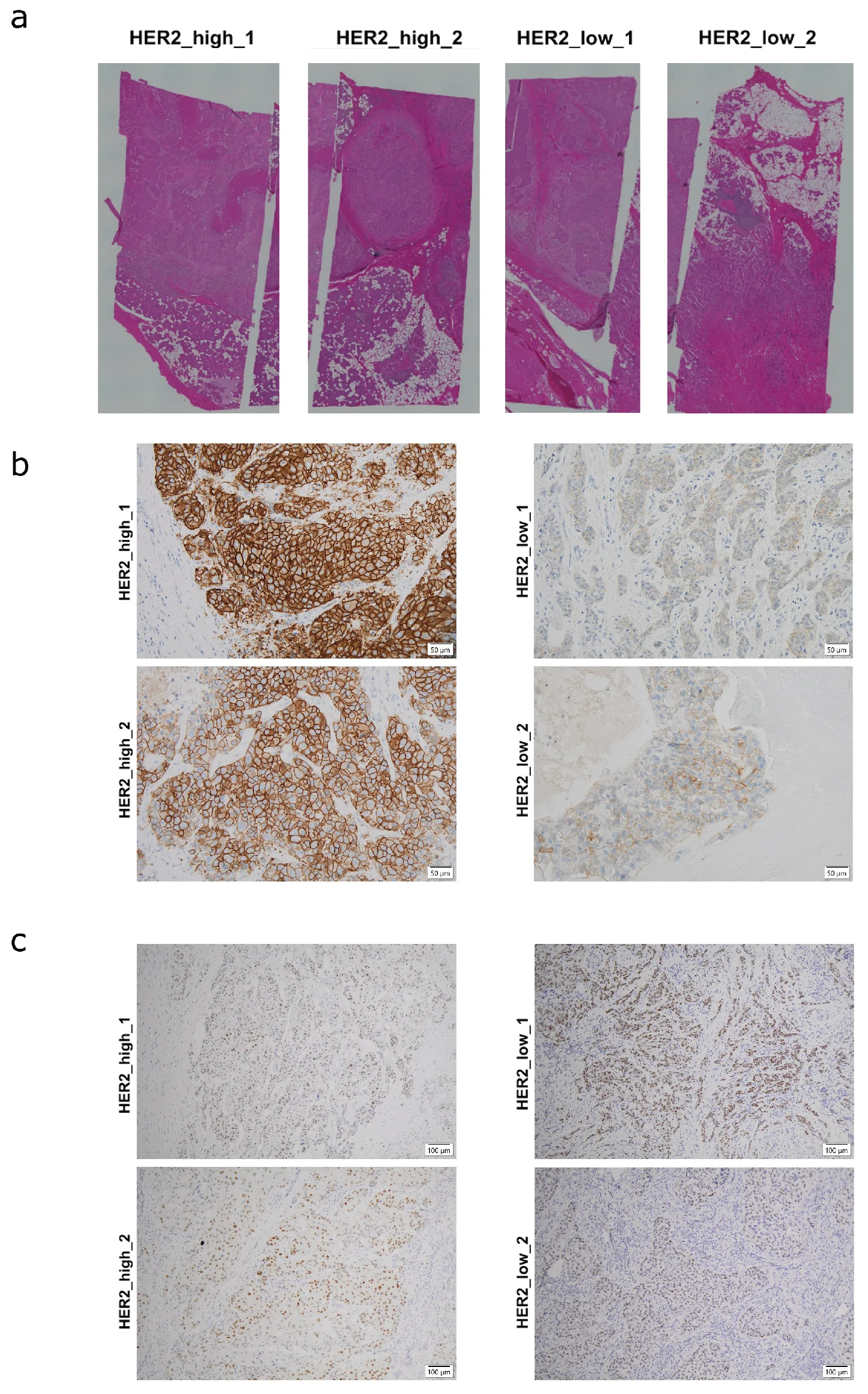
Histological and immunohistochemical features of SDC samples used for spatial transcriptomic analysis. (**a**) Hematoxylin and eosin (H&E)-stained sections of four surgically resected SDC specimens. (**b**) Representative HER2 immunohistochemical (IHC) staining of the four SDC specimens. Two samples exhibited strong HER2 overexpression (IHC 3+), whereas the remaining samples were classified as HER2-low tumors (IHC 1+). (**c**) Representative AR IHC staining of the four SDC specimens. Nuclear AR immunoreactivity was observed in approximately 80%, 80%, 70%, and 80% of tumor cells in HER2_high_1, HER2_high_2, HER2_low_1, and HER2_low_2, respectively.

Four surgically resected SDC specimens originating from the parotid gland were included in this study. Two tumors were classified as HER2-high (HER2 immunohistochemistry [IHC] 3+), whereas the remaining two were confirmed as HER2-low (IHC 1+) upon pathological re-evaluation, despite focal areas of weak complete membranous staining that did not meet the criteria for an IHC 2+ classification. Specimens were designated as HER2_high_1, HER2_high_2, HER2_low_1, and HER2_low_2, according to their HER2 status. All four tumors were AR-positive by IHC, showing clear nuclear immunoreactivity in a substantial proportion of tumor cells. Hematoxylin and eosin (H&E), HER2 IHC and AR IHC images are presented in Figure 1. 

### 2.2. Spatial Clustering and Histological Annotation

**Figure 2:**
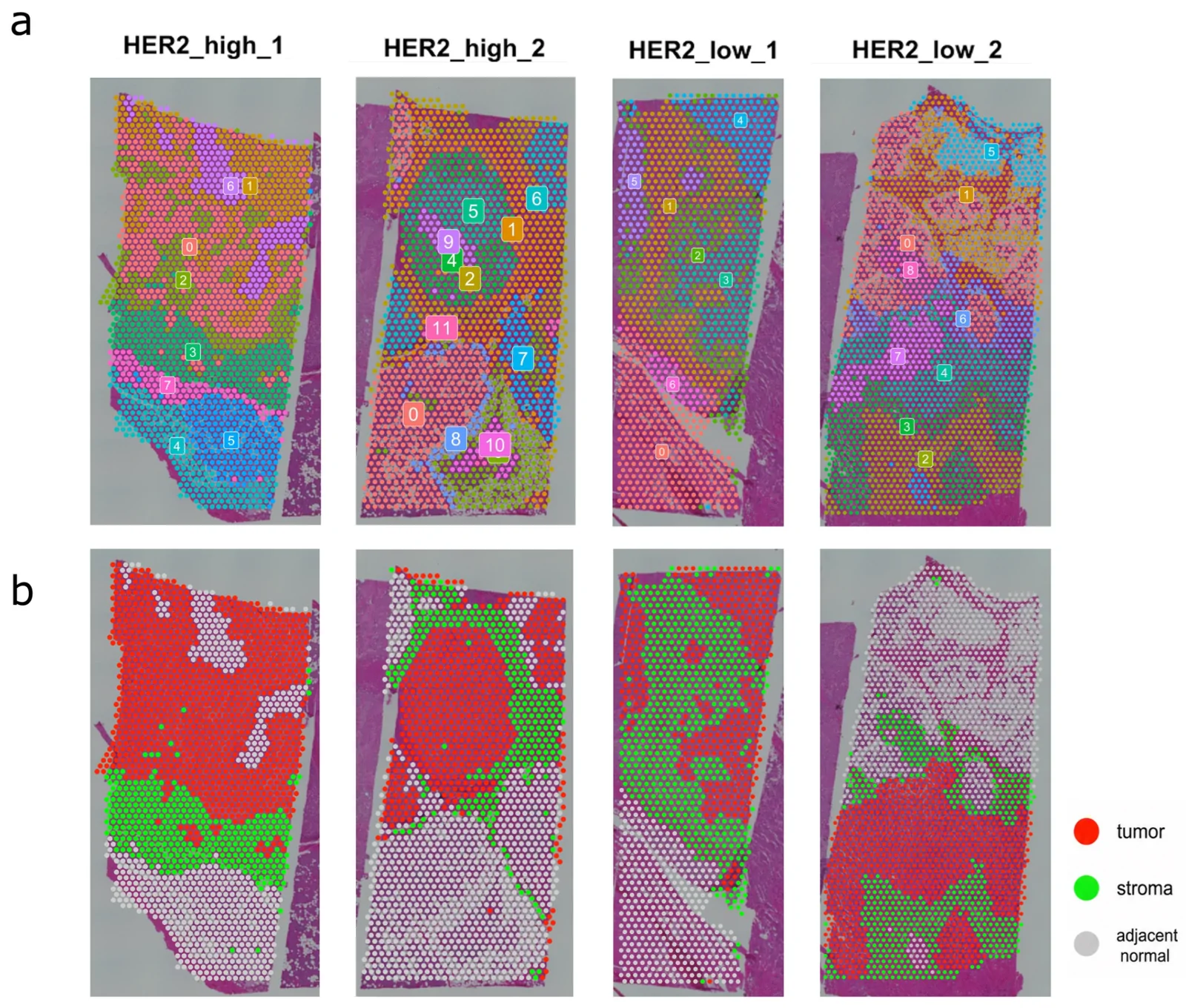
Spatial clustering and histology-guided tissue annotation of SDC samples. (**a**) Unsupervised spatial clustering overlaid on H&E-stained sections, demonstrating correspondence between transcriptomic clusters and histological architecture. Numbers indicate distinct transcriptomic clusters. (**b**) Histology- and transcriptome-guided annotation of spatial clusters into tumor (red), stromal (green), and adjacent normal (gray) regions.

We performed Visium spatial transcriptomic analysis of the four SDC specimens to characterize their spatial gene expression patterns. Unsupervised spatial clustering revealed distinct transcriptomic clusters within each SDC specimen (Figure 2a). Integration of histological morphology and spatial gene expression profiles enabled the classification of these clusters into tumor, stromal, and adjacent normal regions (Figure 2b). 

### 2.3. Identification of HER2- and AR-Associated Tumor States

**Figure 3:**
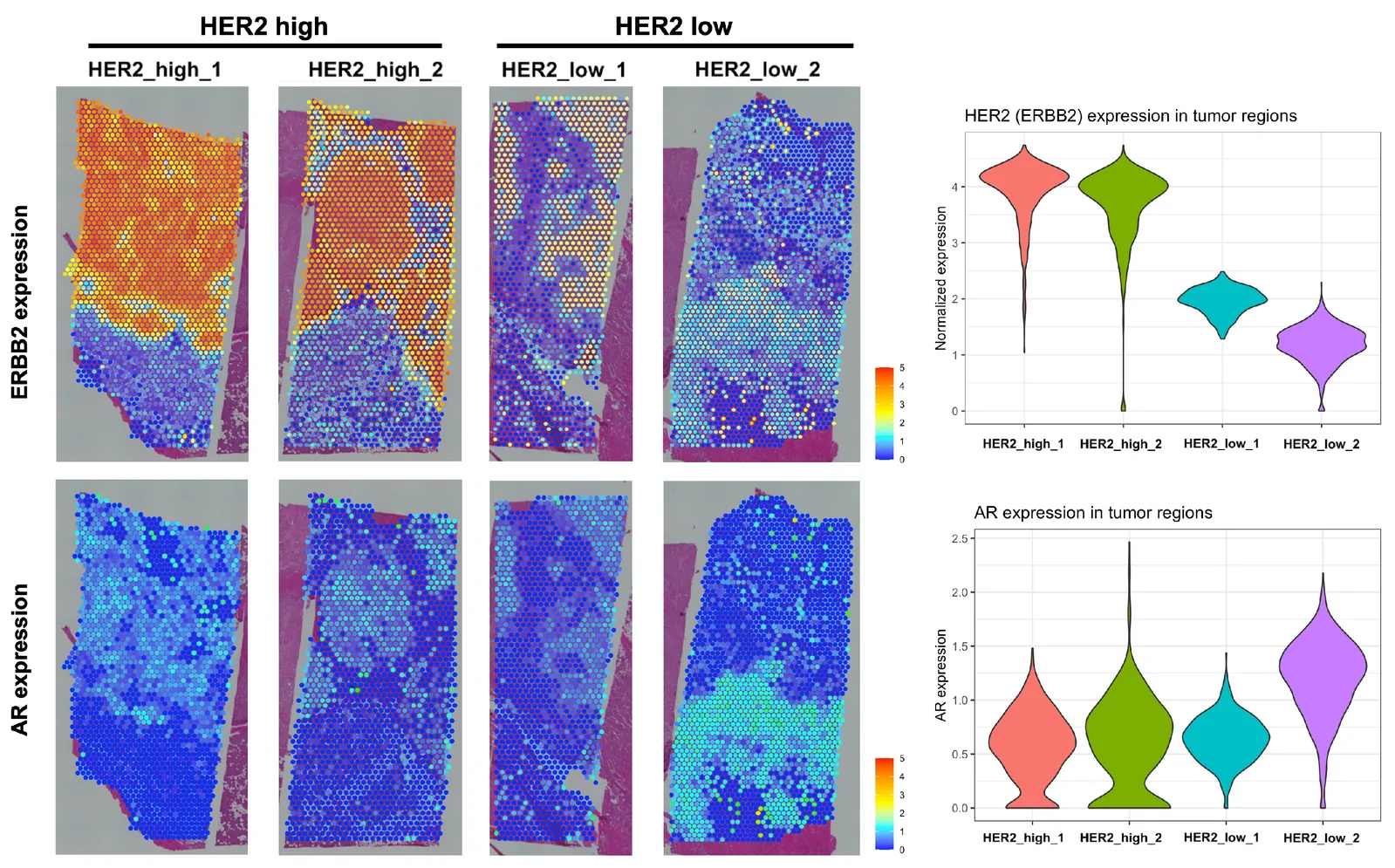
Spatial expression patterns of *ERBB2* and *AR* in SDC tumor regions. Spatial transcriptomic maps and violin plots depicting normalized *ERBB2* and *AR* expression across annotated tumor regions in four SDC samples. *ERBB2* expression was markedly elevated in the two HER2-high (IHC 3+) tumors, consistent with immunohistochemical findings, whereas HER2-low tumors showed substantially reduced expression. *AR* was broadly expressed across tumor regions in all samples, with relatively higher levels observed in sample HER2_low_2.

To characterize intratumoral transcriptional programs associated with HER2 and AR, analyses were restricted to spots annotated as “tumor.” *ERBB2* and *AR* expression were detected across tumor regions in all four samples, although at varying levels (Figure 3). Therefore, we further assessed their associated transcriptional activity to characterize HER2- and AR-associated states within each tumor.

**Figure 4:**
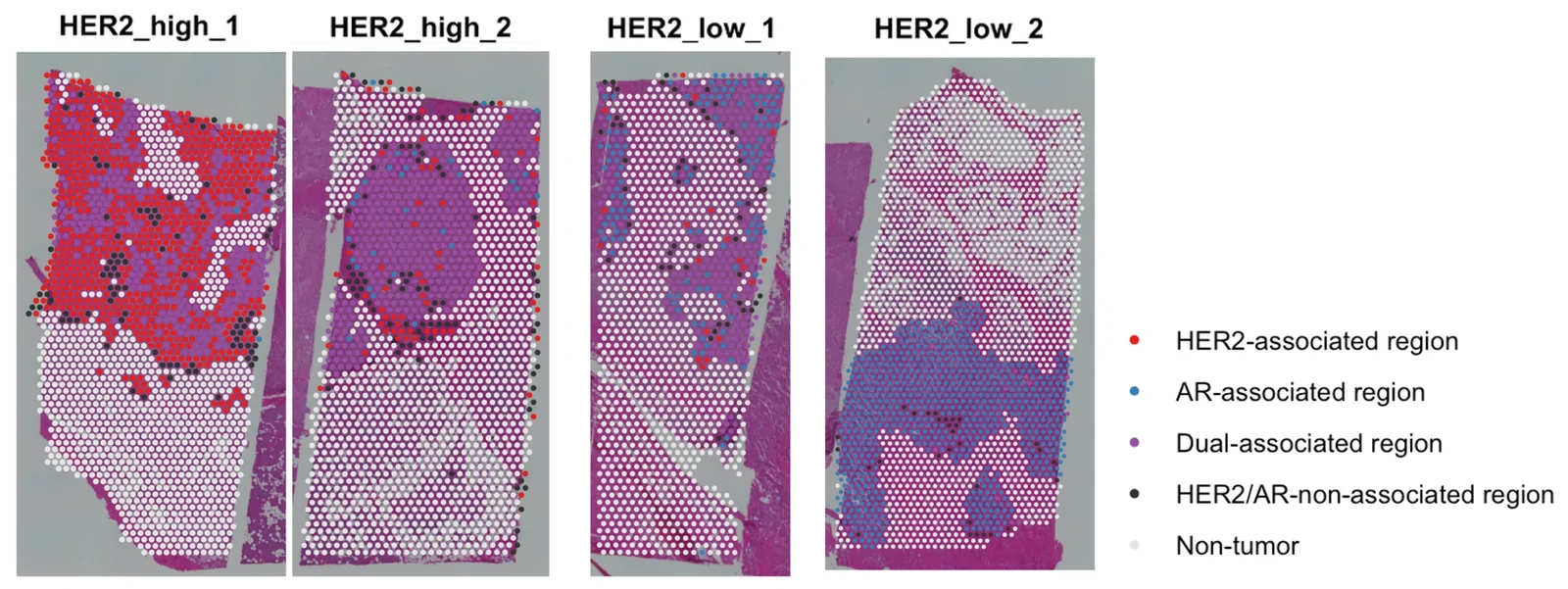
Spatial identification of HER2- and AR-associated transcriptional states in SDC. Spatial classification of tumor spots based on *ERBB2* and *AR* expression and associated transcriptional signature scores. HER2-associated regions are shown in red, AR-associated regions in blue, dual-associated regions in purple, and HER2/AR-non-associated regions in black. Non-tumor regions are shown in white. The predominant spatial states were HER2-associated in HER2_high_1 (63.9%), dual-associated in HER2_high_2 (66.7%), dual-associated in HER2_low_1 (45.1%), and AR-associated in HER2_low_2 (94.1%).

Tumor spots were classified according to HER2- and AR-associated transcriptional states based on *ERBB2* or *AR* expression and their corresponding transcriptional signature scores. Spatial mapping demonstrated heterogeneous distributions of HER2-associated, AR-associated, dual-associated, and HER2/AR-non-associated regions within individual tumors (Figure 4). At the specimen level, HER2 IHC status was broadly consistent with *ERBB2* transcript expression, with higher expression in HER2-high tumors than in HER2-low tumors (Figure 1b and Figure 3). In contrast, although all four tumors showed substantial nuclear AR immunoreactivity and broadly detectable *AR* transcripts (Figure 1c and Figure 3), AR-associated transcriptional states varied among specimens, with HER2-associated regions predominating in HER2_high_1 and AR-associated regions predominating in HER2_low_2 (Figure 4).

### 2.4. Identification and Prioritization of Candidate Surface Therapeutic Targets

**Figure 5:**
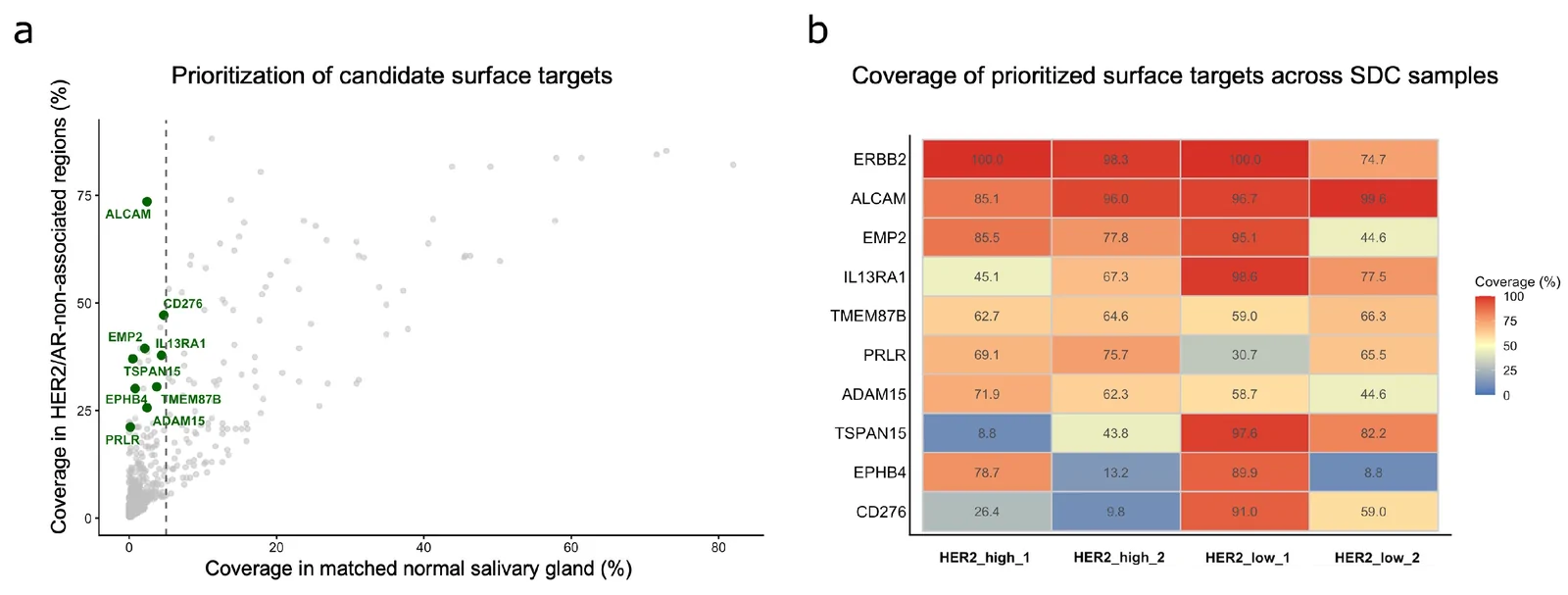
Identification of candidate therapeutic surface targets in SDC. (**a**) Surface-gene coverage in HER2/AR-non-associated and matched normal salivary gland regions. Green points indicate prioritized candidates, and the dashed line indicates the 5% normal salivary gland coverage criterion. (**b**) Whole-tumor coverage of selected surface targets across four SDC samples.

Based on coverage in HER2/AR-non-associated regions, low expression in matched normal salivary gland tissue (≤5% coverage), and therapeutic relevance, nine candidate surface targets were prioritized: *ALCAM*, *EMP2*, *IL13RA1*, *TMEM87B*, *PRLR*, *ADAM15*, *TSPAN15*, *EPHB4*, and *CD276* (Figure 5a). Whole-tumor coverage of the prioritized candidates was subsequently evaluated across the four SDC samples (Figure 5b). Specimen-level mean normalized expression values of the prioritized surface targets are provided in Table S1. Among the evaluated targets, *ALCAM* and *ERBB2* showed consistently high coverage across all four samples (85.1–99.6% and 74.7–100%, respectively).

## 3. Discussion

The management of recurrent or metastatic SDC is currently guided primarily by HER2 and AR expression status. HER2-targeted therapy with trastuzumab plus docetaxel has demonstrated favorable clinical efficacy in HER2-positive SDC [4]; however, its use is generally limited to tumors with strong HER2 overexpression. Therefore, expanding therapeutic strategies for HER2-low or HER2-negative SDC remains clinically important. Likewise, androgen deprivation therapy, including CAB with luteinizing hormone-releasing hormone agonists and antiandrogens, has become an established treatment strategy for AR-positive SDC [5,11]. Although these therapies have shown promising clinical activity, durable responses are not achieved in all patients, and several important clinical questions remain unresolved, including the optimal treatment sequence for tumors co-expressing HER2 and AR. Therefore, in this study, we characterized the spatial heterogeneity of HER2- and AR-associated transcriptional states within SDC. We also explored alternative therapeutic targets in HER2/AR-non-associated regions to identify potential treatment strategies that may complement existing HER2- and AR-directed therapies.

ST analysis demonstrated that HER2- and AR-associated tumor regions exhibited heterogeneous spatial distributions within individual tumors. Tumor-level HER2 or AR expression does not necessarily reflect a uniform transcriptional state throughout the tumor. To the best of our knowledge, this is the first study to characterize the spatial distribution of HER2- and AR-associated transcriptional programs in SDC using ST.

These findings may have important therapeutic implications. The spatial heterogeneity of HER2- and AR-associated transcriptional states suggests that HER2 or AR expression alone may not fully reflect the relative contribution of these programs within individual tumors. Notably, in one HER2 IHC 3+ tumor, HER2-associated transcriptional states predominated despite concurrent AR expression. This finding suggests that AR expression alone may not fully reflect the spatial activity of AR-associated transcriptional programs. This observation may also be relevant to the clinical finding by Kawakita et al., who reported that HER2-targeted therapy was associated with superior response rates and progression-free survival compared with CAB as first-line therapy in HER2-positive/AR-positive SDC [12].

In addition to HER2- and AR-associated transcriptional states, HER2/AR-non-associated regions were identified in all four tumors. Their coexistence with HER2- and AR-associated regions further highlights the intratumoral heterogeneity of SDC and raises the possibility that these regions may contribute to incomplete responses to HER2- or AR-directed therapies. Conceptually, these regions may resemble therapy-refractory tumor states described in other malignancies, including triple-negative breast cancer and AR-independent prostate cancer, in which reduced dependence on canonical oncogenic drivers is associated with limited therapeutic options [13,14]. In these malignancies, therapeutic development has increasingly focused on alternative vulnerabilities, particularly cell-surface targets and antibody–drug conjugate (ADC)-based strategies. We therefore focused on HER2/AR-non-associated regions as a discovery compartment to identify alternative cell-surface therapeutic targets with minimal expression in matched normal salivary gland tissue.

HER2 remains an established therapeutic target in SDC. Notably, our spatial analysis showed that *ERBB2* expression remained broadly detectable even in HER2/AR-non-associated regions, indicating that the absence of a HER2-associated transcriptional state does not necessarily imply loss of *ERBB2* expression. This observation suggests that HER2-targeted ADCs, such as T-DXd, may retain therapeutic activity in tumors lacking strong HER2-associated transcriptional activity. Recent clinical activity of T-DXd in HER2-low SDC in the phase II MYTHOS trial is consistent with this concept [10].

Among the newly prioritized candidates, ALCAM (CD166) showed the broadest and most consistent whole-tumor coverage across all four SDC samples, while maintaining low expression in matched normal salivary gland regions. Although the broad expression of ALCAM in normal epithelial tissues has historically raised concerns regarding on-target, off-tumor toxicity, its low expression in matched normal salivary gland regions in the present study supports its potential relevance as a therapeutic target in SDC. Furthermore, the development of conditionally activated ADCs, such as praluzatamab ravtansine (CX-2009), has demonstrated the clinical feasibility of CD166-targeted therapy in advanced solid tumors [15]. These advances support further investigation of ALCAM-directed therapeutic strategies in SDC.

EMP2 and PRLR also showed relatively broad whole-tumor coverage across multiple samples. EMP2 has recently gained attention as a therapeutic target in several solid tumors [16], supporting its potential relevance in SDC. PRLR has also been explored as a therapeutic target, including the clinical development of PRLR-directed ADCs, providing a precedent for pharmacological targeting of this receptor [17].

In contrast, TSPAN15, EPHB4, and CD276 (B7-H3) showed greater intertumoral heterogeneity. TSPAN15 is of potential interest because monoclonal antibodies against TSPAN15 with inhibitory activity have been developed [18]. EPHB4-targeted CAR-T cell therapy has entered early-phase clinical evaluation (jRCT2033230299) [19], while B7-H3-targeted ADCs and other immunotherapeutic strategies are under clinical development for solid tumors [20]. Thus, despite their heterogeneous tumor coverage, EPHB4 and CD276 may represent translationally relevant therapeutic targets in selected SDCs.

Interestingly, TMEM87B showed relatively consistent whole-tumor coverage across all four samples, despite limited development as a therapeutic target. Although its therapeutic tractability remains to be established, this finding highlights the potential of spatial surfaceome screening to identify less-explored candidate targets in SDC.

The present study has several limitations. First, ST analysis was performed on only four tumor samples, limiting the generalizability of the findings. Moreover, all samples were obtained from primary tumors, whereas systemic targeted therapies are primarily used for recurrent or metastatic disease. Therefore, validation in recurrent and metastatic SDC is required to determine whether the spatial patterns and candidate targets identified here are retained during disease progression. Second, the findings were based primarily on transcriptomic data without comprehensive protein-level validation or functional characterization. Because IHC and ST analyses were performed on non-identical tissue sections, direct spatial correspondence between protein and transcript expression could not be established. In addition, the robustness of the AR-associated spatial classification across alternative androgen-related gene signatures was not formally evaluated in this study. Furthermore, the 55-µm spot-based resolution of conventional Visium precludes definitive assignment of transcriptomic signals to individual tumor cells. Accordingly, the therapeutic relevance of the identified candidate targets requires validation in larger cohorts and functional studies. Nevertheless, this study has notable strengths. The integration of ST with expert histopathological annotation enabled detailed spatial characterization of tumor and stromal compartments while preserving tissue architecture, providing spatial information that is not captured by conventional bulk or dissociative single-cell transcriptomic analyses.

## 4. Materials and Methods

### 4.1. Patient Samples and Histopathological Evaluation

Four formalin-fixed, paraffin-embedded (FFPE) surgical specimens pathologically diagnosed as SDC were obtained from patients treated at Shinshu University Hospital. All tumors originated in the parotid gland. HER2 IHC expression was evaluated by two pathologists according to the 2018 ASCO/CAP guidelines, as previously applied to SDC [9]. Regions for Visium ST analysis were selected through consensus between head and neck surgeons and pathologists to include tumor invasion front and adjacent normal tissue.

### 4.2. ST Analysis

ST analysis was performed using the Visium Spatial Gene Expression platform for FFPE tissue (10× Genomics, Pleasanton, CA, USA) with a 55-µm spot diameter. Tissue processing, probe hybridization, library preparation, and sequencing were performed according to the manufacturer’s instructions, followed by data preprocessing using the Space Ranger pipeline (version 4.0.1; 10× Genomics, Pleasanton, CA, USA). All tissue-associated spots identified by Space Ranger were retained for downstream analysis, and no additional spot-level filtering based on feature or count thresholds was applied. Specimen-specific quality-control metrics, including the number of analyzed spots, mean reads per spot, median genes per spot, and median counts per spot, are provided in Table S2.

Downstream analyses were conducted in R (version 4.5.3; R Foundation for Statistical Computing, Vienna, Austria) using the Seurat package (version 5.5.0). Gene-expression data were normalized using the LogNormalize method with a scale factor of 10,000. Unsupervised transcriptome-based spatial clustering was performed independently for each specimen. Each cluster was subsequently annotated based on integrated evaluation of histological morphology on the corresponding H&E sections and spatial gene expression profiles. Clusters showing epithelial/tumor-associated expression patterns and corresponding histological features were classified as tumor, whereas clusters showing stromal or microenvironment-associated transcriptional patterns were classified as stroma. Clusters predominantly corresponding to adjacent normal salivary gland tissue or other non-neoplastic components were designated as adjacent normal.

### 4.3. Definition of HER2- and AR-Associated Tumor States

To characterize intratumoral transcriptional programs associated with HER2 and AR, analyses were restricted to spots annotated as “tumor.” HER2- and AR-associated transcriptional activity was assessed using curated gene sets from the Molecular Signatures Database (MSigDB) [21]. HER2-associated transcriptional activity was evaluated using the REACTOME_SIGNALING_BY_ERBB2_IN_CANCER gene set, whereas AR-associated transcriptional activity was evaluated using the DOANE_RESPONSE_TO_ANDROGEN_UP gene set. The HER2-associated signature was selected to represent *ERBB2*-related signaling, whereas the AR-associated signature was selected based on previous transcriptomic evidence supporting the relevance of androgen-responsive gene programs in SDC [22]. Signature scores were calculated for individual Visium spots using AddModuleScore in Seurat.

Thresholds were determined separately for each specimen using matched histologically annotated normal salivary gland regions as specimen-specific references. The 95th percentile of *ERBB2* or *AR* expression and the corresponding signature score in matched normal salivary gland spots was used as the threshold for elevated expression and transcriptional activity, respectively. HER2-associated regions were defined as tumor spots in which both *ERBB2* expression and the HER2-associated signature score exceeded their respective thresholds. Similarly, AR-associated regions were defined as tumor spots in which both *AR* expression and the AR-associated signature score exceeded their respective thresholds. Spots meeting both criteria were classified as dual-associated regions, whereas spots meeting neither criterion were classified as HER2/AR-non-associated regions.

### 4.4. Identification and Prioritization of Candidate Surface Therapeutic Targets

To identify alternative therapeutic targets in HER2/AR-non-associated tumor regions, genes were initially ranked according to the proportion of HER2/AR-non-associated spots with detectable expression. Spots with normalized expression values >1 were considered positive, and the proportion of positive HER2/AR-non-associated spots was calculated for each gene.

Candidate cell-surface proteins were identified using the human surfaceome defined by SURFY [23]. Expression in matched histologically annotated normal salivary gland regions was evaluated using the same threshold. As a stringent exploratory criterion to enrich for targets with minimal expression in normal salivary gland tissue, genes expressed in ≤5% of matched normal salivary gland spots were retained for further evaluation.

Candidate cell-surface therapeutic targets were prioritized from highly ranked surface genes based on broad coverage of HER2/AR-non-associated regions and evidence of previous or ongoing development of antibody-based, antibody–drug conjugate (ADC), chimeric antigen receptor (CAR) T-cell therapy, or receptor-targeted therapies. Genes predominantly associated with stromal, vascular, or immune compartments were not prioritized as tumor-directed surface targets.

Whole-tumor coverage of the prioritized candidates was then evaluated separately for each SDC specimen as the proportion of annotated tumor spots with normalized expression values >1. ERBB2 was additionally included as an established therapeutic target for comparison. The complete ranking of candidate surface genes is provided in Table S3.

## 5. Conclusions

ST analysis revealed distinct HER2- and AR-associated transcriptional states within SDC, including HER2/AR-non-associated regions. Surfaceome-based screening of these regions identified several candidate therapeutic targets with low expression in matched normal salivary gland tissue, some of which showed broad whole-tumor coverage. These findings highlight the intratumoral heterogeneity of SDC and provide a rationale for exploring alternative therapeutic strategies that may complement existing HER2- and AR-directed therapies. Further validation, including protein-level analyses and in vitro and in vivo functional studies, is required to establish the clinical utility of these candidate targets.

## Data Availability

The raw and processed spatial transcriptomic data generated in this study have been deposited in the Gene Expression Omnibus (GEO) under accession number GSE349781.

## Supplementary Materials

The following supporting information can be downloaded at: https://www.mdpi.com/article/10.3390/ijms27209018/s1.

## Abbreviations

The following abbreviations are used in this manuscript:

ADC	antibody–drug conjugate
AR	androgen receptor
CAB	combined androgen blockade
CAF	cancer-associated fibroblast
CAR	chimeric antigen receptor
FFPE	formalin-fixed, paraffin-embedded
H&E	hematoxylin and eosin
HER2	human epidermal growth factor receptor 2
HNC	Head and neck cancer
HNSCC	head and neck squamous cell carcinoma
ICIs	immune checkpoint inhibitors
IHC	immunohistochemical
SCC	squamous cell carcinoma
SDC	Salivary duct carcinoma
SGCs	Salivary gland carcinomas
ST	spatial transcriptomics
T-DXd	trastuzumab deruxtecan
